# Indole Alkaloids from *Fischerella* Inhibit Vertebrate Development in the Zebrafish (*Danio rerio*) Embryo Model

**DOI:** 10.3390/toxins6123568

**Published:** 2014-12-22

**Authors:** Katherine Walton, Miroslav Gantar, Patrick D. L. Gibbs, Michael C. Schmale, John P. Berry

**Affiliations:** 1 Department of Chemistry and Biochemistry, Florida International University, 3000 NE 151st Street, North Miami, FL 33181, USA; E-Mail: berryj@fiu.edu; 2 Department of Biological Sciences, Florida International University, 11200 SW 8th Street, Miami, FL 33199, USA; E-Mail: gantarm@fiu.edu; 3 Division of Marine Biology and Fisheries, Rosenstiel School of Marine and Atmospheric Sciences, University of Miami, 4600 Rickenbacker Causeway, Miami, FL 33146, USA; E-Mails: pgibbs@rsmas.miami.edu (P.D.L.G.); mschmale@rsmas.miami.edu (M.C.S.)

**Keywords:** *Fischerella*, Stigonemataceae, cyanobacteria, hapalindoles, ambiguines, indole alkaloids, zebrafish embryo, teratogenicity, vertebrate toxicity, harmful algal blooms

## Abstract

Cyanobacteria are recognized producers of toxic or otherwise bioactive metabolite associated, in particular, with so-called “harmful algal blooms” (HABs) and eutrophication of freshwater systems. In the present study, two apparently teratogenic indole alkaloids from a freshwater strain of the widespread cyanobacterial genus, *Fischerella* (Stigonemataceae), were isolated by bioassay-guided fractionation, specifically using the zebrafish (*Danio rerio*) embryo, as a model of vertebrate development. The two alkaloids include the previously known 12-epi-hapalindole H isonitrile (**1**), and a new nitrile-containing variant, 12-epi-ambiguine B nitrile (**2**). Although both compounds were toxic to developing embryos, the former compound was shown to be relatively more potent, and to correlate best with the observed embryo toxicity. Related indole alkaloids from *Fischerella*, and other genera in the Stigonemataceae, have been widely reported as antimicrobial compounds, specifically in association with apparent allelopathy. However, this is the first report of their vertebrate toxicity, and the observed teratogenicity of these alkaloids supports a possible contribution to the toxicity of this widespread cyanobacterial family, particularly in relation to freshwater HABs and eutrophication.

## 1. Introduction

Cyanobacteria (or “blue-green algae”) are recognized to produce a multitude of bioactive secondary metabolites. A relatively small subset of these compounds have been linked—particularly in association with eutrophication and episodic “harmful algal blooms” (HABs) in freshwater systems—to environmental health concerns including acute poisoning of humans and other animals and putative chronic health effects [1]. As a result of their observed bioactivity, several of these metabolites have been additionally investigated as possible leads for drug discovery [2]. Furthermore, a simultaneously growing body of knowledge is emerging with respect to the potential ecological or otherwise functional role of these metabolites for cyanobacteria [3].

As part of on-going investigations into freshwater cyanobacterial metabolites as “toxins,” the zebrafish (*Danio rerio*) embryo, as a model of vertebrate development, has been previously developed and applied as a means to identify, isolate and characterize bioactive metabolites, and specifically *teratogenic* compounds that inhibit or impair developmental pathways or processes [4,5,6]. Indeed, the zebrafish has more generally emerged as an important model in a wide range of fields including basic (e.g., genetics, developmental biology) and applied sciences (e.g., biotechnology, toxicology/pharmacology, drug discovery) [7,8]. However, owing to several practical aspects associated with zebrafish embryos—including high fecundity (*i.e.*, access to hundreds or thousands of eggs/embryos per breeding), small size (≤1 mm diameter), transparency of embryos and rapid embryogenesis—the early developmental stages of the zebrafish (*i.e.*, 1 to 5 days post fertilization) have, in particular, enabled a wide-range of medium to high-throughput biological, e.g., toxicological, assays. Moreover, as an aquatic animal model, the zebrafish embryo has proven to be a particularly relevant toxicological model for understanding a range of environmental contaminants, including the toxic secondary metabolites from cyanobacteria and other HAB species [5]. In this regard, the zebrafish embryo has been employed in studies ranging from general toxicological characterization [4,5,6,9,10,11,12,13,14,15] of established cyanobacterial toxins, as well as otherwise uncharacterized cyanobacterial metabolites (e.g., components of extracts/mixtures), to studies, such as the present one, which specifically employ the zebrafish embryo model toward isolation and subsequent chemical characterization, of bioactive cyanobacterial metabolites [15,16,17,18].

**Figure 1:**
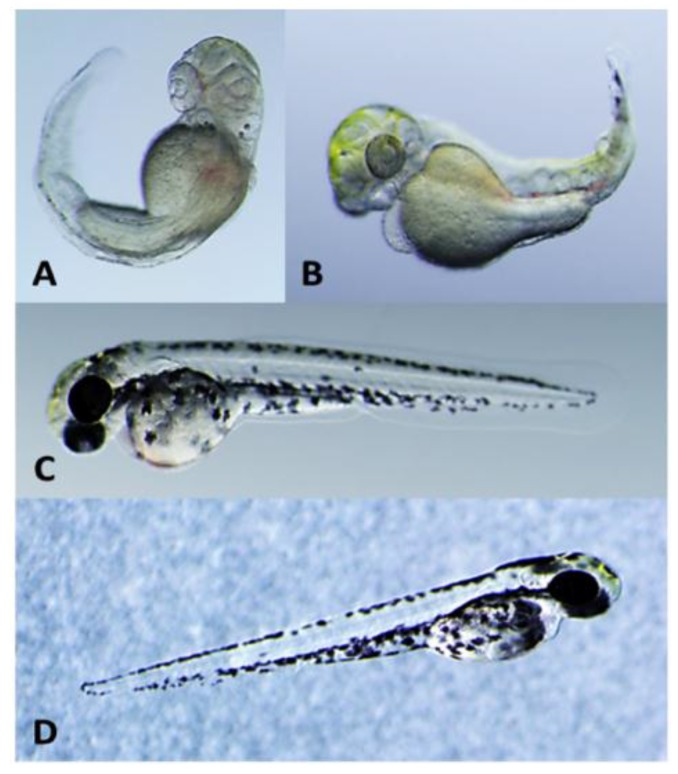
Developmental effects in zebrafish embryos (3 days post-fertilization) exposed to 10 µg/mL of crude extracts from *Fischerella* 52-1 (**A**); **1** (**B**); and **2** (**C**); shown for comparison is a vehicle (*i.e.*, solvent) only control (**D**).

In one such prior study [5], a culture collection of cyanobacteria, specifically isolated from the Florida Everglades and other freshwater sources in South Florida, was screened using the zebrafish embryo as an assay of teratogenicity. In this study, extracts from a strain (52-1) of the cyanobacterial genus, *Fischerella*, were found to exert pronounced activity [5]. In particular, a clear and reproducible pattern of developmental dysfunction, uniquely characterized by lack of pigmentation and/or migration of melanocytes (Figure 1), along with a range of other abnormalities, was observed for embryos exposed to extracts from this cyanobacterial isolate.

In the present study, the zebrafish embryo teratogenicity assay was employed for bioassay-guided fractionation toward purification, and subsequent chemical and toxicological characterization, of the previously identified bioactive metabolites from *Fischerella* 52-1. Using this approach, two indole alkaloids—specifically belonging to a class of compounds characteristic of the genus, and family (Stigonemataceae) more generally—including one previously known variant (**1**), and one apparently novel, nitrile containing congener (**2**), were identified and characterized.

## 2. Results and Discussion

### 2.1. Cyanobacterial Material

*Fischerella* 52-1 was previously isolated from a South-Central Florida lake (Lake Tennessee, Polk County, FL, USA), and established as a unialgal culture. The strain was initially identified, based on morphology, as a member of the genus, *Fischerella*, and subsequent 16s rDNA sequence analysis confirmed this identification. BLAST search of 16s rDNA sequences (see Figure S1 in Supplementary Information; and Experimental Section) suggested the isolate to be either *F. muscicola* or *F. ambigua.* That said, recent taxonomic analyses [19] generally suggest that unambiguous assignments of species epiphets within the genus, and the Stigonemataceae more generally, including those associated with 16s rDNA sequence analyses submitted to GenBank, remain to be adequately resolved.

*Fischerella*, and other members of the family Stigonemataceae, classified as a taxon of “true branching” filamentous cyanobacteria, have been demonstrated to produce a variety of bioactive compounds including alkaloids, polychlorinated aromatic compounds, and cyclic peptides [20,21,22,23,24,25,26,27,28,29,30,31,32,33,34]. However, the arguably most characteristic class of bioactive metabolites isolated from numerous genera within Stigonemataceae is a structurally diverse group of indole alkaloids, including hapalindoles, ambiguines, fischerindoles and welwitindolinones [20,21,24,25,26,27,28,29,30,31,32,33,34] that are not generally known outside of the family. In particular, biological activity of these indole alkaloids has been largely investigated with respect to a antimicrobial (e.g., antibacterial, antifungal, antialgal) activity as it relates to a suggested role in allelopathy [26,27].

### 2.2. Teratogenicity and Identification of Indole Alkaloids from Fischerella 52-1

Exposure of zebrafish embryos to extracts of *Fischerella* 52-1 cultures, immediately following fertilization (*i.e.*, <6 h post-fertilization), resulted in a consistent and reproducible pattern of abnormal development (or so-called “teratotype”) by 3 days post-fertilization (dpf), specifically characterized by a lack of pigmentation and/or migration of melanocytes, and other dysfunctions (Figure 1). Clear similarities between embryos exposed to extracts in the present study and the previously observed [5] teratotype (Figure 1) indicated presence of the same or similar bioactive metabolites in our continuous cultures of the strain. Each component of the culture—including cellular biomass, culture medium and *exudates* that accumulated in culture flasks—was extracted, and shown to be bioactive. However, owing to the quantitatively more pronounced bioactivity, *i.e.*, minimal concentration for observed teratogenicity, of extracts (approximately 25-fold higher on weight per volume concentration basis, *i.e.*, ≥10 µg/mL, compared to biomass and culture medium, *i.e.*, ≥250 µg/mL), and the relatively simpler chemical composition of this fraction of the culture—as well as the potential relevance of extracellular toxins (see below)—isolation focused on the bioactive components of the exudates. In fact, comparison of the chemical composition, by HPLC-UV (see Figure S2 in Supplementary Information), generally indicated the presence of several of the same components, including the two major constituents (**1**,**2**) purified and characterized in the present study, in active fraction from all three components of the culture.

Bioassay-guided fractionation of exudates, based on teratogenicity in the zebrafish embryo, enabled isolation of the two major constituents (**1**,**2**) in bioactive fractions from culture exudates. At each fractionation step, teratogenicity in the zebrafish embryos (exposed to the fractions from the chemical isolation) was assessed to determine which chemical fractions to target for subsequent isolation steps. As described in the Experimental Section, consequent chemical isolation from exudates included methanol extraction, and two steps of preparative HPLC, for isolation of **1** and **2**. This approach identified, in fact, several chemically related metabolites, and specifically apparent indole alkaloids (as evidenced by UV absorbance; see below, and Figure S3 in Supplementary Information) that are characteristic of the genus and family (Stigonemataceae). However, only three of these components showed significant teratogenicity at comparable concentrations (≤10 µg/mL), and only two (**1** and **2**) represented major components (Figure S3 in Supplementary Information). Sequence of citations (Figure S4).

**Figure 2:**
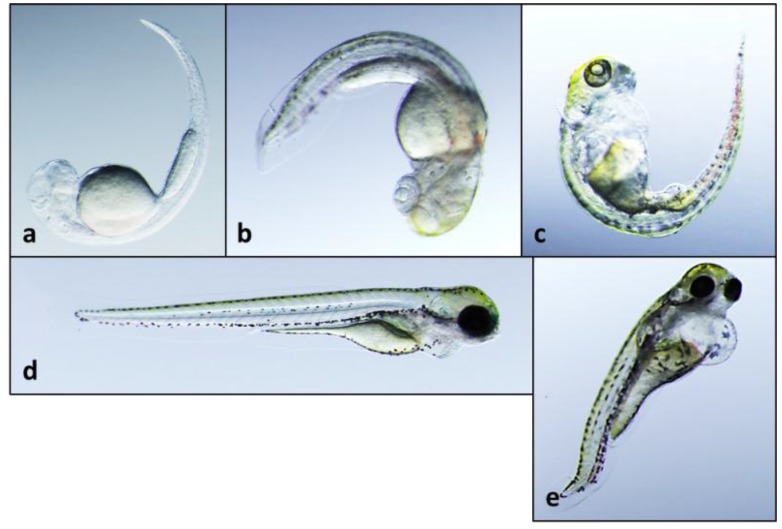
Teratogenicity of **1** tested at 5 µg/mL concentration following exposure for (**a**) 2 days post-fertilization (dpf); (**b**) 3 dpf; and (**c**) 5 dpf; Also shown are 5 dpf embryos with apparent partial recovery from teratogenic effects when embryos removed from treatment after 2 dpf (**d**); and 3 dpf (**e**).

**Figure 3:**
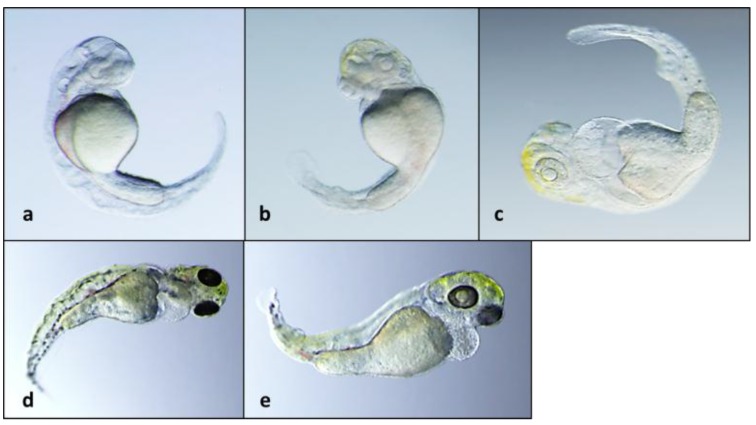
Teratogenicity of **1** tested at 10 µg/mL concentration following exposure for (**a**) 2 days post-fertilization (dpf); (**b**) 3 dpf; and (**c**) 5 dpf; Also shown are 5 dpf embryos with apparent partial recovery from teratogenic effects when embryos removed from treatment after 2 dpf (**d**); and 3 dpf (**e**).

Of the two congeners, **1** showed activity in the zebrafish embryo teratogenicity assay at the lowest concentrations (≥5 µg/mL, *i.e.* ~16 µM) tested, and particularly reproducible effects on development (Figure 1, Figure 2 and Figure 3) that were qualitatively consistent with the previously observed teratotype [5]. In particular, embryos exposed to **1** at 2, 3 and 5 dpf were characterized by overall lack of pigmentation, *i.e.*, melanophores, severe curvature of the body axis and pericardial edema with a dose-dependence of the teratogenic effects (Figure 2 and Figure 3; Figure S4). On the other hand, embryos exposed to **2** were characterized by relatively limited effects on development, and at only the highest concentrations (≥10 µg/mL) tested. For example, although embryos exposed to the lowest active concentration (10 µg/mL, *i.e.*, 47 µM) of **2** were consistently characterized at 3 dpf by a slight curvature of the body axis, relative to controls (Figure 1), these deformities were generally abrogated by 5 dpf (see Figure S5 in Supplementary Information). Notably, a potential for recovery from teratogenicity was observed in embryos exposed to active concentrations (*i.e.*, 5 and 10 µg/mL) of **1** (Figure 2 and Figure 3, respectively) when removed from treatments. Specifically, 5 dpf embryos removed from treatment with **1** at either 2 or 3 dpf indicated clear mitigation of—and even recovery from—effects on development, and potential for recovery was clearly correlated with exposure concentration and consequent severity of the teratogenicity (Figure 2 and Figure 3). Interestingly, the concentrations at which teratogenicity was observed, in each case, are approximately equivalent to the range (*i.e.*, ~10^1^–10^2^ µM minimum inhibitory concentrations) previously observed for antimicrobial activity of indole alkaloids from *Fischerella* [33].

### 2.3. 12-Epi-Hapalindole H Isonitrile

The seemingly more bioactive **1** was purified as an amorphous white solid, and subsequently identified as the previously reported 12-epi-hapalindole H isonitrile [28]. An indole moiety of **1** was suggested absorbance (λ_max_ at 221, 281, and 290 sh) in the UV spectrum as has been observed previously for indole alkaloids isolated from *Fischerella* [20,21,27,28,29,30,31]. Low resolution MS analysis identified a presumptive molecular ion ([M + H]^+^) at m/z 305, as well as a dimer ([2M + H]^+^) and fragmentation product, specifically corresponding to the loss of an isonitrile (*i.e.*, [M–HCN]^+^), at *m*/*z* 609 and 278, respectively. The nominal mass of the [M + H]^+^ of **1**, as well as the presence of an apparent isonitrile functional group, was consistent with the tetracyclic hapalindole class of alkaloids previously isolated from *Fischerella* and related genera [20,21,27,28,29,30,31], and formation of dimers similar to that observed for **1** has, likewise, been observed for other hapalindoles [29]. Moreover, HRMS provided an accurate mass (*m*/*z* 305.24534), and corresponding molecular formula of C_21_H_25_N_2_, consistent with the [M + H]^+^ of a tetracyclic hapalindole [27,28]. Infrared spectroscopic analysis of **1** afforded two unique peaks indicative of a secondary amine (3412.1 cm^−1^) as found in an indole ring, and an isonitrile group (2137.07 cm^−1^) as found in many of the related indole alkaloids reported for *Fischerella* and the Stigonemataceae [9,10,16,17,18,19,20]. Subsequent dereplication based on NMR analysis, including ^1^H- and ^13^C-NMR, as well as COSY, HMQC and HMBC, confirmed the identity of **1** as the previously reported [28] 12-epi-hapalindole H isonitrile (see Table S1 and Figure S6 in Supplementary Information).

### 2.4. 12-Epi-Ambiguine B Nitrile

The second major component of the active fractions (**2**), was isolated as an amorphous white solid, and identified as a member of the tetracyclic ambiguine sub-class of alkaloids [30]. Low resolution MS (*i.e.*, LC-HESI-MS) analysis identified a nominal mass of 422 amu for **2**, specifically giving an [M − H]^−^ ion at *m*/*z* 421 and [M + H]^+^ at *m*/*z* 423 in negative and positive ionization modes, respectively. Moreover, 3:1 ion clusters at *m/z* 423/425 and 421/423 in positive and negative ionization modes, respectively, indicated the presence of a chlorine atom as found in many of the indole alkaloids from the Stigonemataceae [20,24,30]. The exact mass of the molecular ion was determined to be 422.2128 amu, corresponding to a molecular formula of C_26_H_31_OClN_2_. The mass and corresponding molecular formula of **2**, therefore, suggested that it was likely a tetracyclic ambiguine as first isolated, in fact, from *Fischerella* [30]. Fragmentation of the [M + H]^+^ ion produced a number of daughter ions proposed to include [M–CH_3_]^+^, [M–Cl]^+^, [M–H_2_O]^+^, and [M–(2CH_3_ + CN)]^+^ which are, likewise, consistent with an ambiguine, and specifically ambiguine B isonitrile [30]. However, IR and ^13^C-NMR data for **2** (Table 1; see below) lacked the characteristic peak produced by an isonitrile group in this variant [30].

**Table 1:** NMR Spectroscopic Data (400 MHz, C_6_D_6_) for 12-Epi-Ambiguine B Nitrile (**2**).

Position	δ_C_, type	δ_H_ (*J* in Hz)	COSY	HMBC	NOESY
1		7.32, s		9	
2	139.5 C			27, 28	
3	113.4 C				
4	140.9 C			6, 17, 18	
5	115.6 CH	7.10, dd (0.5, 7.3)	6	6, 7	17
6	123.9 CH	7.27, dd (7.3, 8)	5, 7	4, 5	17
7	108.7 CH	6.96, dd (0.5, 8)	6	5, 9	
8	132.9 C				
9	126.3 C			7	
10	73.9 C	1.54, OH		11	11,18, 27, 21*E*
11eq	54.4 CH	4.13, s		10, 12, 13, 15, 19, 20, 23	27, 28
12	44.9 C			11, 19, 21	
13ax	67.4 CH	4.16, dd (4.4, 12.6)	14ax, 14eq	11, 19	15
14ax	30.5 CH_2_	2.65, q (12.5)	13, 14eq, 15ax		18, 20
14eq	30.5 CH_2_	2.25, ddd (2.2, 4.3)	13, 14ax, 15ax		15, 17
15ax	52 CH	2.45, dd (2, 12.6)	13, 14ax, 14eq	11, 17, 18	13, 17
16	37.6 C			17, 18	
17	27.2 CH_3_	1.28, s		4, 15, 16	5,6
18	27.7 CH_3_	1.18, s		4, 15, 16	
19	27.6 CH_3_	1.58, s		11, 12, 13, 20	11, 13, 21*E*
20	142.7 CH	6.78, dd (11, 17.6)	21*E*, 21*Z*	11, 19	14ax
21*E*	114.5 CH_2_	5.05, dd (1, 17.6)	20, 21*Z*	12	19
21*Z*	114.5 CH_2_	5.09, dd (1, 11)	20, 21*E*	12	
23	120.1 C			11	
24	39.5 C			26, 27, 28	
25	148 CH	6.05, dd (10.5, 17.6)	26*E*, 26*Z*	27, 28	
26*E*	113.3 CH_2_	5.00, dd (1, 17.6)	25, 26*Z*	24	27, 28
26*Z*	113.3 CH_2_	4.95, dd (1, 10.5)	25, 26*E*	24	
27	29.4 CH_3_	1.05, s		2, 24, 25	11
28	28.1 CH_3_	1.26, s		2, 24, 25	11

**Figure 4:**
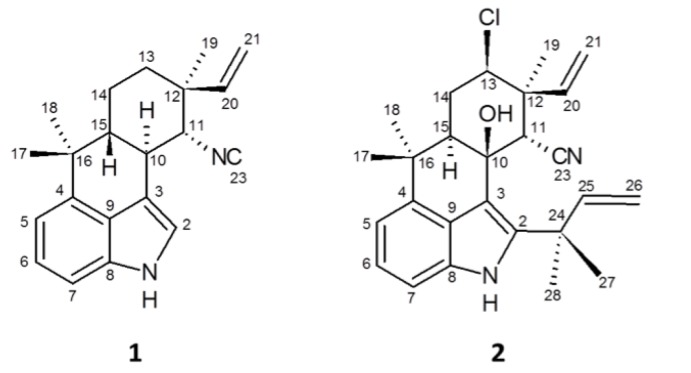
Chemical structures of 12-epi-hapalindole H isonitrile (**1**) and 12-ambiguine B nitrile (**2**) isolated from *Fischerella* 52-1 based on teratogenicity in the zebrafish embryo model.

To further characterize **2**,^1^H- and ^13^C-NMR, as well as COSY, HMQC, HMBC and NOESY were performed (Table 1; Figure 4). ^1^H- and ^13^C-NMR revealed that **2** contained 31 hydrogens and 26 carbons (Table 1), consistent with the molecular formula proposed by HRMS, and tentative identification as ambiguine B isonitrile [30]. Moreover, comparison of NMR data to previously reported ambiguines revealed the presence of several features similar to those found in ambiguine B isonitrile, including 2 terminal alkenes, 5 methyl groups, an indole moiety *C*-bonded at C-2, C-3, and C-4, a C-10 hydroxyl group and a cyclohexane ring. However, notable exceptions were specifically observed with respect to C-11, -19, and 23, and to a lesser extent C-20 and C-21 (Table 1). Accordingly, it was suggested that **2** represents a potentially new ambiguine variant. In particular, the expected chemical shift (δ_C_ 159), corresponding to an isonitrile group, was not observed for **2**, and in conjunction with the lack of the signal in the IR (as discussed above), it was accordingly concluded that **2** did not contain an isonitrile.

**Figure 5:**
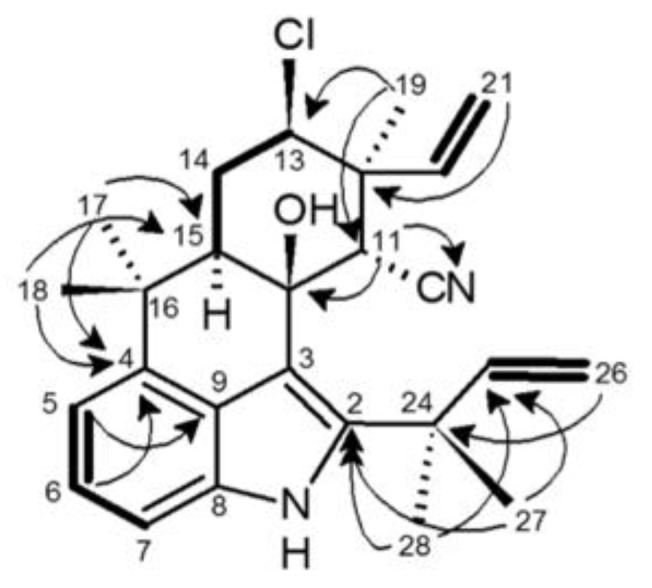
Key HMBC (arrows) and COSY (bold line) correlations of 12-epi-ambiguine B nitrile (**2**).

The relative configuration of the cyclohexane ring was primarily deduced based on coupling constants and NOESY correlations (Table 1). The proton on the chlorine-bearing C-13 (tentatively assigned based on comparison to published data for ambiguines [29]) coupled to H-14_eq_ and H-14_ax_, and based on calculated coupling constants (4.3 and 12.5 Hz, respectively), which were nearly identical to those reported for ambiguine B isonitrile [30], was determined as axial. The 14eq/14ax protons coupled, in turn, to a doublet of doublets (δ_H_ 2.45) that was similarly identified as the axial proton on C-15 based on both comparison to chemical shifts reported for ambiguine B isonitrile [30], and coupling constants (2.2 and 12.6 Hz, respectively, for H-14_eq_ and H-14_ax_). The proton at the C-11 position (δ_H_ 4.13) was assigned based on comparison to the previously reported (and approximately similar) chemical shift for isonitrile-bearing alkaloids [30], HMBC correlations (Table 1; Figure 5) and observation as a singlet consistent with this proton being isolated from adjacent protons. The hydroxyl group tentatively assigned to C-10 (based on comparison to published data [30]) was subsequently shown, in NOESY analysis, to correlate to two methyl groups, the doublet of doublets (δ_H_ 5.05) assigned to the proton on C-21*E*, and the proton on C-11. Thus, from this analysis it was determined that the hydroxyl group was axial as this position would afford spatial correlation to at least two methyl groups, one equatorial hydrogen on C-11, and either of the terminal alkene protons depending on the stereochemistry. Finally, NOESY was used to propose the relative configuration of the terminal alkene assigned to C-20 and C-21 position. Specifically, H-14_ax_ correlated with the hydrogen on C-20 consistent, therefore, with a C-12 epimer as observed for **1** and other indole alkaloids from the genus [27,28]. Further supporting this relative configuration, a singlet (δ_H_ 1.58) assigned as the C-19 methyl group was shown to correlate in NOESY to the C-13 proton, the singlet assigned as the proton on C-11, and the doublet of doublets assigned as C-21*E* proton (Table 1).

When compared to the previously reported alkaloids, the chemical shifts of all carbons closely matched ambiguine B isonitrile with the exception of C-11, -19, -20, -21, and -23. The discrepancy for C-19, C-20 and C-21 was attributed to the proposed orientation of the substituents on C-12, and corresponding assignment as the 12-epimer of ambiguine B. On the other hand, the discrepancy between **2** and ambiguine B isonitrile with respect to C-11 and C-23 was attributed to the yet unidentified functional group on C-11. In fact, after the assignment of all other carbons, only one was left unassigned. The unassigned carbon was determined in DEPT experiments to be quaternary. In addition, the formula produced from the mass spectrometric analysis specified two nitrogens, and only one had been assigned to the structure in the indole moiety. With this information many possible functional groups were eliminated; however, the chemical shift of this quaternary carbon (δ_C_ 120.1) very closely matched that of nitriles previously reported in other indole alkaloids from *Fischerella* [34], and **2** was consequently concluded to be 12-epi-ambiguine B nitrile.

Although quantitatively and qualitatively less active than **1** in the zebrafish embryo assay, the identification of **2** as a nitrile-bearing 12-epi-ambiguine represents a new variant. The vast majority of indole alkaloids previous isolated from *Fischerella* and other members of the Stigonemataceae are characterized as either isonitrile- or isothiocyanate-bearing alkaloids. That said, there have, in fact, been previous reports of nitrile-containing variants from the Stigonemetaceae, including members of the fischerindole and pentacyclic ambiguine sub-classes [32,33,34]. Although not common, these variants further highlight the biosynthetic diversity within this class of compounds, and the relatively lower toxicity of the nitrile congener in the present study—compared, for example, to the isonitrile bearing **1**—supports a possible role of the variable functional group associated with C-11 (along with others, e.g., chlorination at C-13) in the bioactivity of these compounds.

## 3. Experimental Section

### 3.1. General Experimental Procedures

UV spectroscopic data were obtained with Shimadzu Prominence HPLC system equipped with a photodiode array detector. IR spectroscopy was performed on a Perkin Elmer Spectrum IR (Perkin Elmer, Waltham, MA, USA), using solid aliquots of each compound. NMR analyses were performed on a Bruker 400 MHz NMR (Bruker, Billerica, MA, USA), and experiments included, ^1^H-NMR and ^13^C-NMR, DEPT-135, COSY, HMQC, HMBC, and NOESY. The analyses were performed using either *d*-benzene for both, or *d*-methanol for **2** and *d*-methanol and a small amount of D_2_O for **1**. The chemical shifts were calibrated using the methanol solvent shift in the *d*-methanol samples, and 0.05% TMS in the *d*-benzene. Mass spectrometry was performed on two UPLC systems coupled to either an OT Velos Orbitrap Mass Spectrometer (for high-resolution mass), or a Thermo TSQ Quantum triple-quad mass spectrometer (Thermo Scientific, Waltham, MA, USA). UPLC separation for MS analysis was achieved using either 0.1% formic acid in an acetonitrile/water gradient (high-resolution MS) or methanol/water gradient (low resolution MS) with a Phenomenex Kinetex reverse-phase (C-18) column (2.6 µm particle size) (Phenomenex, Bland, MO, USA); ionization, in each case, was achieved by heated electrospray ionization (HESI).

### 3.2. Cyanobacterial Material

*Fischerella* 52-1 was isolated (August 2002) from Lake Tennessee (Polk County, FL, USA) by standard techniques [35]. The isolate was taxonomically identified (to genus) by microscopic observation using classical morphological criteria given in Komarek and Anagnostidis [36]. Subsequent sequencing of 16s rDNA, and subsequent BLAST search, determined the species as either *F. ambigua* or *F. muscicola* (see Figure S1 in Supplementary Information). The 16s rDNA sequences for the isolate were previously submitted to GenBank.

The isolated strain was cultured as previously described [5,6,26]. Briefly, non-axenic unialgal cultures (isolated by filtration on 0.45 µm membrane filters, and selection on BG-11/1.5% agar plates) were grown in aerated 3 L Erlenmeyer flasks at room temperature (24 °C) under continuous light (25 μE/m^2^/s) in BG11 medium, buffered with 2-morpholinoethanesulfonic acid (MES) at pH 7.2, to obtain sufficient material.

### 3.3. Extraction and Isolation

Using the zebrafish embryo teratogenicity assay to guide fractionation, **1** and **2** were isolated from exudates present in cultures that forms as a generally insoluble, whitish material accumulating at the periphery of the surface layer of the cultures. For extraction, this material was collected, by gentle scraping, to remove from culture flasks. Subsequently, the collected material was extracted by an optimized method using two overnight extractions (on a shaker) in methanol. Components from the pooled methanol extracts, following concentration *in vacuo*, were subsequently isolated by a two-step HPLC method, using a Shimadzu Prominence HPLC system equipped with a photodiode array detector (λ_max_ 220–225 nm), and Luna semi-prep C18 column (5 µm particle size, 250 mm × 10 mm; Phenomenex, Torrance, CA, USA). The first method employed a gradient of acetonitrile/water (50%–99% from 0 min to 20 min), followed isocratic 99% acetonitrile 20–30 min), at a flow rate of 4.5 mL min^−1^. Bioactivity was observed for components eluting between 15 min and 20 min, and this combined fraction was further separated using a gradient of MeOH/water (73.2%–73.5% over 40 min, followed by 73.5%–75% from 40 min to 52 min; flow rate = 4.5 mL min^−1^); the major bioactive components (**1** and **2**) were purified as peaks eluting at 42 and 45 min, respectively.

12-epi-Hapalindole H Isonitrile (1): amorphous white solid; UV (PDA) *λ*_max_ 218, 278, 290sh nm; IR *v*_max_ 3412 and 2137 cm^−1^; ^1^H NMR and ^13^C NMR (see Table S1 and Figure S6 in Supplementary Information); HESIMS *m/z* 278 ([M – HCN]^+^), 305 ([M + H]^+^), 609 ([2M + H]^+^); HRHESIMS *m/z* 305.24534 [M + H]^+^ (calculated for C_21_H_25_N_2_, 305.20177).

12-epi-Ambiguine B Nitrile: amorphous white solid; UV (PDA) *λ*_max_ 222, 279, 290sh nm; IR *v*_max_ 3378 and 1616 cm^−1^; ^1^H NMR and ^13^C NMR (see Table 1); HESIMS *m/z* 421/423 (3:1 [M − H]^−^ ion cluster) and *m/z* 423/425 (3:1 [M + H]^+^ ion cluster); HRHESIMS *m/z* 422.2128 [M]^+^ (calculated for C_26_H_31_OClN_2_, 422.21249).

### 3.4. Zebrafish Embryo Toxicity Assay

The zebrafish (*Danio rerio*) embryo, as a model vertebrate development, and specifically indicator of teratogenicity, was employed for bioassay-guided fractionation/purification, and subsequent toxicological characterization, of **1** and **2**. The zebrafish embryo teratogenicity assay was performed as previously reported [5]. Briefly, for each exposure, test compounds or extract/fraction were added, along with relevant solvent controls (*i.e*., equivalent volume of solvent without compounds) were added to wells of a 24-well polypropylene plate (Evergreen Scientific, Los Angeles, CA, USA). Following evaporation of solvents, 1 mL of E3 medium [37] was added to each well. Subsequently, five embryos—specifically selected from 4-cell to 32-cell stage embryos (<6 h post fertilization)—were transferred to each well where they were exposed (over the course of up to 5 days post fertilization [dpf]) to treatments, *i.e.*, extracts, fractions, compounds and solvent controls. Embryos were observed using a dissecting microscope over the course of 5 dpf. All breeding and bioassays involving zebrafish were conducted under protocols approved by FIU and UM Institutional Animal Care and Use Committee (IACUC), and performed by trained investigators.

## 4. Conclusions

Indole alkaloids from the *Fischerella* and other members of the Stigonemataceae have been previously associated, in particular, with biological activity (*i.e.*, antimicrobial, antialgal) related to possible ecological roles, and specifically allelopathy [26]. However, this is the first report of vertebrate toxicity, and specifically teratogenicity, of this class of compounds. The presence of the metabolites in the exudates of cultures does, indeed, further support a possible ecological role of these compounds in allelopathy, but the observed vertebrate toxicity, likewise, suggests a possible contribution of these extracellular alkaloids to the environmental toxicity of the genus. Indeed, *Fischerella*, and the Stigonemataceae, more generally, are relatively widespread in freshwater systems, and these finding suggest that this class of metabolites—characteristic of the taxonomic family—may warrant further consideration as possible HAB toxins.

## Supplementary Materials

Supplementary materials can be accessed at: http://www.mdpi.com/2072-6651/6/12/3568/s1.
